# Effect of Delivery Format on Student Outcomes and Perceptions of a Veterinary Medicine Course: Synchronous Versus Asynchronous Learning

**DOI:** 10.3390/vetsci8020013

**Published:** 2021-01-20

**Authors:** Regina M. Schoenfeld-Tacher, David C. Dorman

**Affiliations:** Department of Molecular and Biomedical Sciences, College of Veterinary Medicine, North Carolina State University, Raleigh, NC 27607, USA; david_dorman@ncsu.edu

**Keywords:** online learning, student perceptions, emergency remote teaching, toxicology

## Abstract

The COVID-19 pandemic prompted instruction at many veterinary schools to switch to an emergency remote teaching format to prevent viral transmission associated with in-person synchronous lectures. This study surveyed student perspectives and academic performance in a pre-planned online second-year veterinary toxicology course given at North Carolina State University in Spring 2020. This course relied on asynchronous narrated presentations for content delivery. This method of delivery predated the pandemic and was used throughout the course. Academic performance and patterns of access to materials in the online course was compared with the access patterns and performance of students given classroom-based synchronous teaching in Spring 2019. Assessments evaluated in this study were identical across courses. Students’ academic performance was unaffected by delivery method. Lack of instructor interaction was an important perceived barrier in the asynchronous course. Asynchronous course materials were uniformly accessed across all days of the week, while supplemental materials for the face-to-face course showed a weekly pattern. Moving from letter grades to pass/fail did not change access frequency to supplemental course materials but led to decreased video usage in the asynchronous course. Results suggest that although some veterinary students perceived the switch in delivery format negatively, the method of delivery did not adversely affect performance in this preclinical course.

## 1. Introduction

The efficacy of distance education in science and veterinary curricula has long been a topic of investigation [1], with multiple studies aiming to assess the effects of online delivery on student learning and classroom interactions [2,3,4,5,6]. Investigations into the feasibility of these techniques to counteract faculty shortages in veterinary medical education and analyses of the cost/benefit to online approaches have been reported since the late 2000s [7,8]. The Journal of Veterinary Medical Education dedicated an issue to this topic in 2007. Despite this growing interest in online education, most veterinary curricula have retained a traditional, didactic mode of instruction, where the students and instructor meet in a fixed location for a defined period of time each week (i.e., synchronous instruction). Synchronous learning fosters real-time feedback and interactions between the instructor and participants. Synchronous learning may take place in a face-to-face environment, with all participants being in the same physical location, or it may occur online, via a virtual platform or videoconferencing technology. Asynchronous learning results when the instructor and learners are not engaged in the learning process at the same time and real-time interactions with other people are absent. There are several advantages to asynchronous learning since participants can learn on their own time and schedule. A meta-study examining the effect of delivery timing within distance education found greater student achievement with asynchronous teaching than classroom instruction [9]; while a separate study employing random assignment found that there was no statistically different performance on course exams for students in an online or face-to-face section of a course [6]. A meta-analysis of online education in medical education [10], found no evidence for enhanced effectiveness of face-to-face instruction on medical students’ knowledge, skills, or retention of material. Thus, it is reasonable to conclude that online instruction should not pose an inherent danger to veterinary students, in terms of learning gains.

Student motivation and engagement are additional factors to consider in evaluating teaching methods. In a study of undergraduate students, Nennig et al. [2] found no significant difference in attitudes towards chemistry among students taking an online or face-to-face course. When work habits of students taking an introductory physics course were compared regarding amount and frequency of access to course resources, there were very few differences across the online and face-to-face sections [3]. However, students in the online section of the course were more likely to access content pages than those in the face-to-face course. This study also demonstrated that students who access materials less frequently (i.e., once per week) had lower exam scores, while those who accessed the materials daily performed better, due to spaced repetition. This effect was stronger for the online group.

Preclinical training of veterinary students at North Carolina State University (NCSU) College of Veterinary Medicine (CVM) has historically relied on a traditional, face-to-face approach, where instructors and students meet synchronously in the same physical location. At NCSU-CVM, opportunities for asynchronous learning and review of content information are also available for large lecture presentations. Most classroom-based didactic sessions in the preclinical curriculum are recorded via Mediasite and made available to students for later viewing through a learning management system (LMS). This allows students to change the time and location in which they access the material, effectively converting synchronous, face-to-face instruction into asynchronous, online delivery. Studies involving medical students have shown that students find these recorded lectures useful and help improve learning [11,12]. This hybrid approach abruptly changed at NCSU-CVM and other veterinary schools in response to the COVID-19 pandemic [13]. Starting in March 2020, all NCSU-CVM preclinical instruction moved to remote online instruction, with a mix of synchronous and asynchronous activities being provided to veterinary students. This shift in instructional delivery also occurred elsewhere in the United States, affecting undergraduate, veterinary, and other medical educational programs [13,14]. The abrupt move to online instruction during the course of a semester can best be characterized as “emergency remote teaching”, with most faculty needing to rapidly convert their face-to-face courses to online delivery [15]. To meet this challenge, many instructors replicated traditional instructional methods, broadcasting didactic lectures via video. This abrupt change, along with all the other events associated with the COVID-19 pandemic, such as stay at home orders, undoubtedly caused a great degree of stress to undergraduate [16,17] and veterinary [18] students. Thus, while this paper focuses on academic outcomes, it is important to consider the backdrop of factors affecting students’ motivation and ability to study during the second half of the 2020 Spring semester.

Unfortunately, the rapid response required to reduce the spread of the SARS-CoV-2 virus at the NCSU-CVM precluded a prospective evaluation of the impact changes in delivery of course material had on either veterinary student perception or academic performance. This study however helps to fill this data gap. In 2019 one of the authors (D.C.D.) was a recipient of a Fulbright-Saastamoinen Foundation Grant in Health Sciences that would allow them to teach and perform research in Finland during part of 2020. This author teaches a core veterinary toxicology course in the second-year veterinary curriculum and a decision was reached between the instructor, department head, and Associate Dean and Director of Academic Affairs that this veterinary toxicology course would be delivered remotely, using asynchronous techniques in the Spring 2020 semester. The advance notice allowed for purposeful design and development of a planned online course. This manuscript describes student performance on several assessments used in the course and compares the performance of the planned online 2020 class with the previous Spring semester when a synchronous, face-to-face delivery methodology was used.

Our study evaluated the following research question: How does conversion of a veterinary toxicology course from a synchronous, face-to-face delivery method to asynchronous online delivery affect students’ academic performance and satisfaction? We explored this question by assessing the following domains:

- Academic performance on course assessments, as an indicator of content mastery;
- Student interaction with online materials, as an indicator of their engagement with the course content;
- Correlation between performance on course assessments and interaction with materials for the online course, as a proxy for effort;
- Changes in interaction with online materials in response to alterations in grading scheme (letter-graded vs. pass/fail);
- Association between anticipated and experienced barriers, as well as changes in attitudes towards distance education after experiencing the course.

## 2. Materials and Methods

### 2.1. Institutional Review Board Approval

The study was conducted in accordance with the Declaration of Helsinki. The protocol was approved as “exempt” by the Institutional Review Board (IRB) Committee of North Carolina State University for use of human subjects (IRB protocol # 20640). All participants were recruited from the 2019 and 2020 Veterinary Toxicology and Poisonous Plant courses given at the NCSU-CVM. An invitation (recruitment materials) was sent via Qualtrics to all students enrolled in the veterinary toxicology course given in 2019 and 2020. The invitation for the 2020 course participants contained a link to the full survey, while the survey for the 2019 course participants only contained the consent form for use of their grade information. All subjects gave their informed consent before accessing the survey.

### 2.2. Description of the Veterinary Toxicology Courses Given in 2019

The required 2-unit Veterinary Toxicology and Poisonous Plant course at the NCSU-CVM is offered annually in the Spring semester. In 2019, this course was delivered using classroom-based synchronous lectures. Lecture materials presented in 2019 were organized around mid-term and final in-person multiple-choice exams (each exam contributed 100 points of a total of 325 points). A total of 24, 50-min lectures were given in the course (Table S1). Lectures were equally divided between the start of the course and the mid-term exam (n = 12 lectures) and between the mid-term and the final exam (n = 12 lectures).

Each lecture was recorded using Mediasite and these video recordings were made available to all students for delayed viewing via the course home page on the NCSU LMS system. Other materials that were made available on the course LMS website included pdf copies of all PowerPoint presentations, study guides for both the mid-term and final exams, and PowerPoint tutorials on both plant identification and dose calculations.

The paper-based multiple-choice exam format used three items (A, B, C) and students were allowed to choose 33 questions from 38 total. The remaining five questions are “skipped” without penalty. In addition, any exam items with an overall performance rate of < 50% were deleted prior to calculating exam scores. Other assessments used in the toxicology course included completion of an online dose calculation quiz worth 25 points, a take home quiz on veterinary diagnostic toxicology (50 points), and preparation of an email response to a mock client (50 points). The email assignment has been previously described in detail [19]. A portion of a lecture was dedicated to this assignment, presenting the major communication concepts associated with the exercise (medical literacy, readability calculations).

### 2.3. Description of the Veterinary Toxicology Courses Given in 2020

In 2020, the veterinary toxicology course was purposely converted to an asynchronous, online format. Narrated Powerpoint slides were used and each course topic (n = 111) ranged in length from 00:03:25 to 0:34:30 hh:mm:ss. The course was divided into three major segments: required materials assessed by the mid-term examination; required materials assessed by the final exam; and supplemental materials that generally provided either an introduction to the course and instructor, overview of systems toxicology (e.g., Introduction to neurotoxicology), or agents that were not included on examinations (Table S2). There were also four special topic videos that discussed the assessments used in the course. One of these topics was dedicated to the e-mail assignment and provided instruction regarding written client communication.

Design of the course paralleled the 2019 course and similar lecture material was assessed by the two major examinations. Total time associated with the required narrated Powerpoint presentations was slightly shorter (18:40:30) when compared to lecture time for the seated class (20 h). All lecture materials including an unedited transcript of each presentation and take-home assignments were made available to students prior to the start of the semester. Study guides and access to other supplemental materials described above were also made available to participants in the 2020 course. No other LMS features (i.e., discussion boards, self-assessments) were used in either version of the course. Assessments used in the 2020 course up to, but not including, the final exam were identical to those used in the face-to-face course given in 2019. The COVID-19 pandemic resulted in cessation of all in person examinations and a change to satisfactory, marginal, unacceptable (SMU) grading on March 24, under emergency circumstances. Thus, the format of the 2020 final exam differed from that used in 2019 and these data are excluded from our analysis.

### 2.4. Perception Survey

All students enrolled in the 2020 (n = 103) offering of the veterinary toxicology course were e-mailed a link to a Qualtrics-based survey. To minimize potential perceptions of coercion and maximize participation, the survey was sent to all students after the course was completed and final grades had been posted.

The following information was collected from all participants: gender, age, prior experience with online courses in college (undergraduate or graduate), number of online courses they had taken prior to the veterinary toxicology course and whether any were in a science, technology, engineering, or mathematics (STEM) discipline. The survey also asked about students’ concerns in regard to the online veterinary toxicology course.

One survey question posed the following: think back to your feelings and concerns prior to beginning the online toxicology course. How concerned were you about the following issues, as they related to your participation in an online veterinary medicine course? The following seven issues were: computer and technical problems could impact access to course materials; unreliable home internet could impact access to course materials; course format could decrease interaction with fellow classmates; course format may not result in a positive learning experience; course format would limit access to the instructor; course format would require active learning; and course format would require discipline and initiative. Another survey question asked the following: now that you have completed the online toxicology course, how big of a barrier did these issues represent? The same issues were used with slight modification to address word tense (e.g., computer and technical problems impacted access to course materials). Two additional survey questions asked students to reflect on their initial and final impressions concerning distance education. The first question asked: before you started this course, how well did you agree with each of the following statements? While the second asked: after completing the course, how well do you agree with each of the following statements? The following eight statements were then provided for these questions: distance learning provides more motivation for acquisition of knowledge; there is no difference in the quality of knowledge acquired by distance learning; distance learning provides the possibility of independent evaluation; distance learning provides independence of time and place for instruction; distance learning requires possessing special skills to work on the computer; face to face contact is necessary for acquiring and mastering the material; distance learning provides faster and easier mastery of the material; and face to face contact is necessary for interactions with other students in the course. A five-point Likert scale (not at all = 1, slightly, somewhat, moderately, extremely = 5 was used for each of these questions.

Other survey questions included:

- *Before starting the course, how did you expect the workload associated with this online course (Toxicology, 2 credits) to compare with that of a traditionally delivered 2 credit course (e.g., physiology, pharmacology)?* Survey responses were limited to: this course would require more effort; they would require the same amount of effort; or this course would require less effort.
- *After completing the course, how did the workload associated with this online course (Toxicology, 2 credits) actually compare with that of a traditionally delivered 2 credit course (e.g., physiology, pharmacology)?* Survey responses were limited to: this course required more effort; they required the same amount of effort; or this course required less effort.
- *Now that you have completed the course, how do you feel your class as a whole performed in this course (average course grade), as compared to the performance of last year’s class (DVM 2021)?* Survey responses were limited to: much better; better; the same; worse; or much worse.

### 2.5. Data Analysis

Video data were reported as hours:minutes:seconds. Descriptive analyses were performed on the demographic data. Statistical significance for all analyses was associated with *p* < 0.05. Differences in the pre-/post- retrospective survey were analyzed via a Wilcoxon signed ranks test. We assessed differences in performance on tests and assignments between the two groups (students enrolled in the 2019 or 2020 courses) using an unpaired *t*-test. Mean Likert scores were calculated based on the following numerical scale: not at all (value = 1), slightly (2), somewhat (3), moderately (4), and extremely (value = 5). Unless otherwise noted, we report mean (± standard deviation (SD)) values. Access to online materials was examined by reviewing LMS (Moodle) and video (Mediasite) server logs and downloading data for statistical analysis. Use of LMS materials was measured as number of clicks on course items per day. Unpaired *t*-tests were used to compare total daily number of accesses for the online and face to face courses, as well as for the periods when the online course was letter-graded vs. pass/fail. One-way ANOVA was used to analyze differences in access patterns for each course, based on day of the week. Data from the Mediasite server contained access information for each user and video. Descriptive data are presented for the number of accesses per video. Unpaired *t*-tests were used to compare the total number of accesses to videos per day during the periods when it was letter graded vs. pass/fail, as well as the total number of accesses to videos designated as required vs. optional. A one-way ANOVA was used to compare total number of accesses for videos associated with the mid-term exam, final-exam and optional topics. A separate ANOVA was conducted to assess for differences in access frequency based on day of the week. A Pearson correlation was used to explore the relationship between the total amount of time an individual user spent watching the required videos with their score on the mid-term assessment.

## 3. Results

### 3.1. Class Performance on Common Assessments Used in the 2019 and 2020 Toxicology Courses

Table 1 presents the mean (± SD) academic performance scores for the seated (2019) and distance education (2020) courses. Students enrolled in the 2020 toxicology course that relied on asynchronous teaching scored higher on the diagnostic toxicology quiz when compared with students enrolled in the face-to-face synchronous course offered in 2019. The lowest performing students (i.e., bottom 2.5%) enrolled in 2020 performed lower than their counterparts in the seated course given in 2019; however, the number of students (n = 3) preclude any formal analysis (lowest overall course score face-to-face class = 75.8; online = 69.7).

### 3.2. Response to Survey Questions by Students Enrolled in the 2020 Online Toxicology Course

A total of 44 students who were enrolled in the 2020 veterinary toxicology course completed a portion of the survey, resulting in a 43% response rate. Their mean age was 25.1 ± 3.0 years and the survey respondents included 17.5% males, 80.0% females, and 2.5% non-binary (n = 40). The gender distribution is representative of the overall population of veterinary students. Among survey respondents, 87.5% had prior experience with an online course (n = 40). The mean number of online courses taken was 2.5 ± 1.4 courses (n = 34). The majority (91.2%) of students with prior online experience took an online STEM course (n = 34). The majority of students (52.6%) recalled thinking that the online toxicology course would require an equivalent amount of effort to a comparable traditionally delivered course—whereas 13.2% thought the course would require more effort and 14.2% thought less effort would be required (n = 38). After completing the course 23.7% of students felt the course required more effort; 39.5% felt it required the same amount of effort and 36.8% felt that the course required less effort than a comparable traditionally delivered course. The majority of students felt that their class would perform the same (52.6%) or better (15.8%) than students enrolled in the 2019 course. Only 7.9% of students thought that the overall performance of the 2020 class would be much worse than that of the 2019 class.

Table 2 provides the student’s level of concern regarding how different factors may have affected their access to course materials, interactions with peers, or the instructor. Students were moderately concerned that the online course required discipline, initiative, and active learning, the course format could have a negative impact on interactions with their peers and the instructor, and could negatively impact their learning experience. Concerns related to lack of internet or computer access to view the course materials were not identified as an important barrier. When pre- and post-test results were compared, these technology-related concerns demonstrated a statistically significant decrease after the course, within the realm of slightly to somewhat concerned. While students initially had moderate concerns about the effect online delivery would have on their access to the instructor, this level of concern appeared to diminish after the course, moving from moderately to somewhat.

Table 3 provides the students level of agreement with statements that related to their motivation as learners, quality of knowledge, skills needed for distance learning and other factors. Students only slightly agreed that distance learning was more motivating for acquisition of knowledge and that there is no difference in the quality of knowledge acquired by distance learning when compared with traditional lectures. They moderately agreed that distance learning provided them with the opportunity to select the time and place for instruction. While the initial level of endorsement for “distance education requires special skills to work on the computer” was low, it was rated even lower after completion of the course, remaining in the realm of slightly to somewhat agree. No statistically significant change in the pre- and post-test level of agreement with the survey questions were observed for any of the other statements.

### 3.3. Access Patterns for Online Materials and Videos

#### 3.3.1. LMS Materials

**Figure 1:**
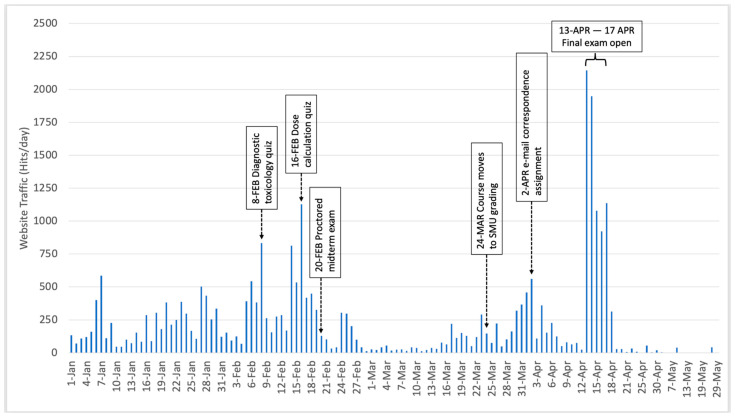
Total number of hits per day on course learning management system (LMS) site for online toxicology course, along with corresponding milestone events. Note that Spring Break occurred 14–22 March 2020. SMU: satisfactory, marginal, unacceptable.

**Figure 2:**
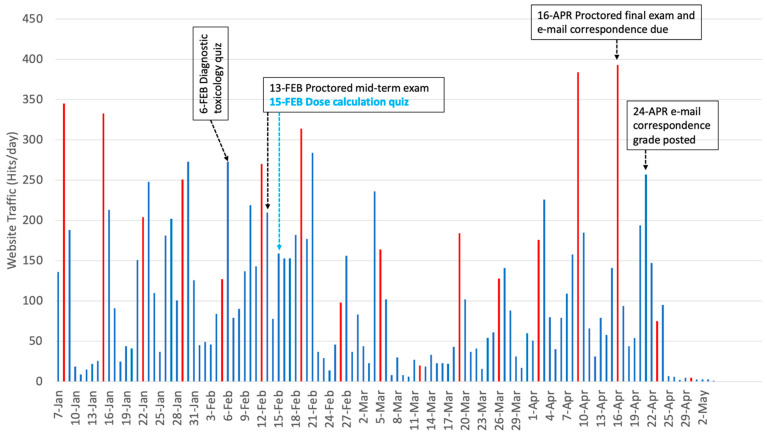
Total number of hits per day on course LMS site for the face-to-face course toxicology course, along with corresponding milestone events. Red lines indicate Tuesdays when maximum access to the course materials occurred. Note that the *y*-axis scale does not match that used in Figure 1.

The LMS used at NCSU, Moodle, tracks student engagement based on hits or clicks on course items. It does not track the amount of time spent by users on any specific page, because elapsed time is not a reliable indicator of activity on a static page. Thus, we compared the number of hits per day for each course, from 1 January through 30 May of the corresponding year. As expected, students had a significantly higher rate of access (t_(152.387)_ = −3.923, *p* < 0.000) to the LMS materials when the course was offered online (mean = 220.6 hits per day) than when it was given face to face (mean =104.3 hits per day). Figure 1 and Figure 2 show the total number of hits per day, for both the online and face-to-face courses, respectively. When traffic for the online course was compared for the time it was letter graded vs. after the move to SMU grading, there no significant difference in access rates (t_(55.936)_ = −0.565, *p* = 0.575), showing that the change in grading policy announced on 24 March 2020 had no effect on usage of the LMS materials. There was no significant access pattern based on day of the week for the online course, as measured by a one-way ANOVA (F_(6,123)_ = 0.495, *p* = 0.81). In contrast, there were significant differences in access patterns for the face-to-face class (F_(6, 110)_ = 8.40, *p* < 0.000). Tukey HSD post-hoc tests revealed that the majority of accesses took place on Tuesdays. As shown in Figure 1 and Figure 2, there was increased traffic in both courses on days when assignments were due. The higher traffic in the online course corresponds to the days the online final exam was available. 

#### 3.3.2. Video Access

**Figure 3:**
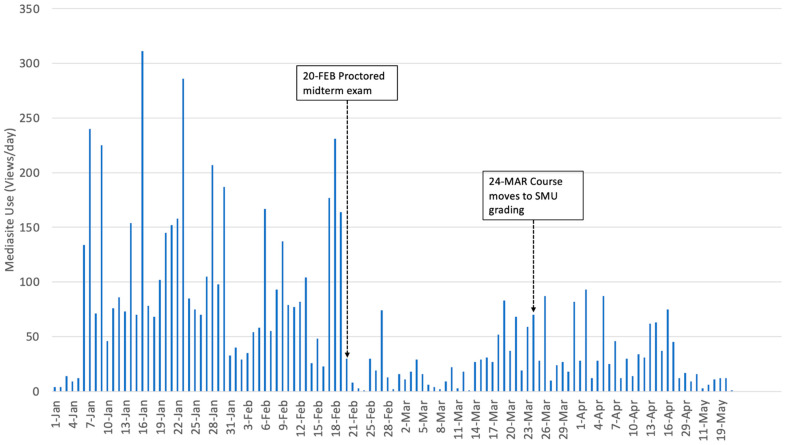
Total number of hits per day on the Mediasite server, for access to videos (narrated Powerpoints) in the online toxicology course, along with corresponding milestone events. Note that Spring Break occurred 14–22 March 2020.

Mediasite access data for both the face to face and online courses were examined in a similar manner. Video recordings of the face-to-face lectures were minimally utilized. Only one participant viewed a total of 90 s of video. Thus, no further analysis was conducted. As expected, the video recordings for the online course received much heavier traffic (Figure 3). The 103 students enrolled in the online course generated a total of 6911 views across the 113 course videos (two organizational elements, plus 111 course topics). Each user accessed a mean of 68 different videos (median = 64, SD = 29). The number of views per user ranged from seven to 170, indicating that some students may not have watched all the course videos, while others watched some videos multiple times. A total of 23:59:50 h of video footage was available for the course topics, with 08:56:40 corresponding to topics assessed on the mid-term, and 09:43:50 corresponding to topics assessed on the final. Each student viewed an average of 12:20:45 h of video footage (median 11:17:21, SD 06:18:25). The total amount of time spent watching the videos ranged from 00:36:59 to 29:05:27. Students spent an average of 7:48:00 watching videos associated with the mid-term exam, which is equivalent to 87% of the content. During the second half of the course, students spent an average of 05:32:36 watching videos associated with the final exam, which is equivalent to 57% of the content.

Due to the manner in which repeated accesses to the same video by a specific user were recorded by the server, we were only able to examine the date a person first watched a particular video. The Mediasite video recordings for the online course received a mean of 59.75 hits per day (median = 34, SD = 62.93). Access rates ranged from zero to 311 hits per day. Peak usage occurred on 16th January, well in advance of the graded mid-term on 20th February. The total number of video accesses per day was significantly higher from 1 January through 23 March, (t_(114.216)_ = 4.185, *p* < 0.000) while the course was letter graded (mean 71.02) than when it was SMU graded, from 24 March through 30 May (mean 33.34).

Video topics associated with the mid-term exam received a mean of 99.58 views by unique users (median 103.5, SD = 17.56), while those associated with the final exam received a mean of 42.3 views by unique users (median = 41.0, SD = 13.88). Videos designated as optional received an average of 15.8 views (median = 13, SD = 11.2). A one-way ANOVA (F_(2,108)_ = 200.29, *p* = 0.000) confirmed that the access rates across all three types of materials were significantly different, with students accessing materials associated with the mid-term most often, and optional materials least often. The most frequently viewed video was associated with absorbents and cathartics, receiving 131 views. There was no significant difference in use of the videos based on day of the week for the online course, as measured by a one-way ANOVA (F_(6,110)_ = 1.48, *p* = 0.19).

### 3.4. Relationship between Video Access and Assessment Performance

The total amount of time each participating user spent watching the video presentations as recorded in the Mediasite access logs, was correlated with their score on the course mid-term assessment. There was no correlation between the amount of time spent watching videos for topics assessed on the mid-term exam and performance on this exam (*r*_(37)_ = 0.134, *p* = 0.42).

## 4. Discussion

While the use of online materials as supplements to classroom based lecture courses is not new to veterinary education [20,21], the planned conversion of full courses to online delivery was rare prior to the onset of COVID-19. Classroom-based lectures remain a prevalent teaching format in medical and veterinary education [12]. Despite this historical reliance on synchronous in-person lectures, there is a growing trend in medical education towards online learning [22]. Multiple studies have examined online instruction in medical education [23,24]. A recent meta-analysis provides no evidence that traditional classrooms were superior to online teaching of undergraduate medical students [10]. The first aim of our study was to determine whether this would also be the case for veterinary students enrolled in a veterinary toxicology course. Similar to reports with undergraduate and medical students, we found that the academic performance of students enrolled in the online veterinary toxicology course was equivalent or superior compared to students receiving course content via a more traditional approach. One advantage of online courses is that they can easily be used across institutions. This may be especially important for veterinary toxicology [25] and other clinical disciplines with a shortage of specialists available to teach the discipline in veterinary schools [8].

Most veterinary students handled the change to a wholly online course well and few encountered significant obstacles. Overall performance on course assessments was comparable to or better than the analogous assessments in the face-to-face version. However, our data indicate that only a small number of individuals (one to five people) found the change to a wholly online format challenging. These students identified concerns related to internet connectivity, lack of computer skills, and other technical issues as possible impediments to success. Decreased classmate and the instructor interactions were also noted as a concern by 13 students. Finally, 14 students perceived the online course to lack the face-to-face contact needed with an instructor to support mastery of course materials. Faculty and administrators should be aware that some individual students, especially those new to online course work may need additional support in meeting the subject matter content outcomes. Sharing the results of this study can provide some reassurance to students, as far as feasibility of mastering course content outcomes in an independent manner.

A variety of frameworks have been proposed to assess the quality of online education [26]. These frameworks often consider access, learning effectiveness, student satisfaction, faculty satisfaction, and cost effectiveness. Our results in regard to student academic performance address one dimension of these criteria, namely that online learning was as effective as learning face to face. Our survey addressed several of these other criteria. For example, we found that many of the students enrolled in the NCSU-CVM curriculum have had prior experience with online education during their undergraduate training. Many of the surveyed students also reported that anticipated concerns regarding computer and technical problems including home internet and their impact on accessibility to course materials decreased after course completion. This change might signal that the materials were in fact easy to access. It could also be the case that students gained technological skills over the course of the semester, as they grew more proficient with online instruction in general. Although the majority of students did not identify these as major impediments to learning, some students (approximately 5%) reported that technical issues had a moderate or extreme negative impact on their ability to access course materials. This low rate of student concerns regarding technical impediments may reflect that the university made computing equipment (e.g., laptops) available for long-term borrowing by students in need. The CVM provided additional emergency financial aid for students needing to obtain internet access or computing equipment. Online technical support was also made available to students experiencing difficulties with their equipment. One drawback to the approach used, narrated PowerPoint lectures, was that it decreased student–student and the potential for student–faculty interactions. Another drawback to the course design was that it lacked two of the key elements crucial for distance education [27]; learner–learner interactions and learner–instructor interactions. However, since these elements were not explicitly built in to the 2019 face-to-face course, it is difficult to assess how the absence of spontaneous interactions affected the online students. Despite these limitations, we did not identify a negative impact on the student’s motivation to learn or master course content.

As expected, students recognized that participation in the online course would require increased discipline and initiative on their part. The instructor provided a tentative class schedule for students enrolled in the online course. This course schedule provided a weekly time schedule that paralleled the schedule used in the seated course. Students also identified the ability to encounter course materials on their own time and place as a positive attribute of the course, and this perception was supported by the data obtained, showing consistent, regular access to the course website. Students’ initial concerns about lack of access to the instructor were often reported to be lower after completion of the course. The instructor also provided three Zoom-based office hours during the semester held during the time designated for the course had it been seated. Participation at these events was sparse (zero to three students). In addition to the instructor’s efforts to provide virtual office hours and monitor e-mail, other factors, such as his early return to the Eastern time zone (due to COVID-19) as well as the emergency migration of all courses to an online format, may have decreased the real and comparative impact of delayed e-mail response times. Within the realm of online courses, asynchronous courses might be particularly beneficial to students in professional programs, as they allow for a more even distribution of workload and spaced practice. The latter assumption was borne out by the access patterns found in this study. Students in the online course demonstrated more even use of the course materials, whereas students in the face-to-face version were more likely to use the materials on a specific day of the week. Based on this observation, we could perhaps speculate that the more frequent access might have contributed to the improved performance of the online students. It is also interesting to note that the evenly distributed access to the LMS materials (but not the videos) was sustained, even after the toxicology course transitioned from letter grades to SMU. This could be an indication of consistent student motivation. However, the observed decrease in video usage may have been related to the assessment format. Instead of watching the videos, students may have opted to research the answers to exam questions while taking the assessment, since it was changed to open-book format. Alternately, students may have opted to rely on the video transcripts to obtain course information.

Another factor that needs to be considered involves faculty involvement in the development of an online course. Primary barriers to developing online courses include faculty time and technical knowledge [28]. Production and editing of the narrated PowerPoint lectures used in the course was time consuming and required development of new materials. In addition, most of the lectures were less than 15 min in duration in keeping with best practices [29]. One disadvantage of this approach was the relative inability to incorporate multiple forms of media into the presentations. Multimedia approaches have been shown to enhance the quality of online medical education [30].

There are several important limitations to the current study. Our response rate was relatively low, and our results are based on a single course conducted at one U.S. veterinary school. Furthermore, students’ perceptions of the online course were likely affected by external stressors associated with the COVID-19 pandemic. Another limitation is that we chose to use a retrospective pre-/-post survey design, where data were collected at the same point in time, regarding participants’ feelings before and after the asynchronous online course. This type of design provides an accurate estimate of treatment effects [31,32], and controls for the response shift bias that occurs in pre- post- test assessments associated with program evaluation [33]. Response shift bias happens when a participant uses a different frame of reference when assessing their feelings or perceptions regarding a particular intervention. We were concerned that a typical pre- post-test design would over-estimate the difference between perceived barriers to online course delivery vs. the actual course experience, as well as underestimate the benefits of this instructional technique [34]. The retrospective pre-/post- study design has been validated for evaluating learning in multiple medical education settings [35,36,37,38,39]. Due to the nature of this study, we assumed that participants truly watched the videos they accessed, as opposed to simply letting them play unattended. Additionally, due to the social distancing measures in effect during the pandemic, we assumed that each user logged in individually, and only watched videos accessed under their credentials. Our study primarily focuses on the ability of students to master subject matter content, as that was the bulk of the material delivered in this pre-clinical toxicology course. Although this course contributed to professional skills development via the client e-mail assignment our results may not be applicable to other courses where interactive communication or other professional or clinical skills are taught. Finally, the change from traditional letter grades to SMU grading precluded quantitative comparisons of academic achievement on the final examination, and also likely affected students’ motivation to learn the content. The veterinary toxicology course will again be offered online in Spring 2021, using a mix of synchronous and asynchronous activities with pass–fail grading. A future study could evaluate whether inclusion of synchronous activities alters student performance or attitudes. Other potential studies could include exploring the effect of online delivery for skills attainment in courses such as communication, where the skills can be practiced outside of a laboratory.

## Figures and Tables

**Table 1:** Student performance on course assessments. All assessments used were identical in both 2019 and 2020 courses. Bold text indicates a statistically significant difference (*p* < 0.05, unpaired two-tailed *t*-test).

Assessment	Point Value	2019 (Face to Face Course)	2020 (Online Course)
Dose calculation quiz	25	24.3 ± 1.7	24.2 ± 1.5
Diagnostic toxicology quiz	50	45.4 ± 2.1	47.9 ± 2.2
Mock client email	50	46.4 ± 2.3	46.4 ± 2.5
Midterm exam	100	92.9 ± 5.6	91.6 ± 6.4
n		98	102

**Table 2:** Mean Likert scores associated with the following survey questions: how concerned were you about the following issues, as they related to your participation in an online veterinary medicine course? and Now that you have completed the online toxicology course, how big of a barrier did these issues represent? Survey prompts have been modified to account for word tense. Bold text indicates a statistically significant difference. Median values are provided in parentheses. A five-point Likert scale (not at all = 1, slightly = 2, somewhat = 3, moderately = 4, extremely = 5) was used for each of these questions.

Survey Prompt	n	Initial Level of Concern	Final Level of Concern	*p*
Computer and technical problems and impact on accessibility to course materials	38	1.7 ± 1.1 (1)	1.4 ± 0.9 (1)	0.008
Unreliable home internet and impact on accessibility to course materials	38	1.8 ± 1.3 (1)	1.5 ± 0.9 (1)	0.015
Negative impact of course format on interaction with my fellow classmates	38	2.9 ± 1.4 (3)	2.7 ± 1.4 (3)	0.319
Course format effect on a positive learning experience	38	3.5 ± 1.3 (4)	3.3 ± 1.5 (3)	0.191
Course format effect on access to the instructor	37	3.2 ± 1.4 (3)	2.8 ± 1.6 (2)	0.019
Course format requires active learning	38	2.7 ± 1.4 (3)	3.0 ± 1.5 (3)	0.192
Course format requires discipline and initiative	38	3.7 ± 1.4 (4)	4.0 ± 1.4 (5)	0.131

**Table 3:** Mean Likert scores associated with the following survey questions: before you started this course, how well did you agree with each of the following statements? and after completing the course, how well do you agree with each of the following statements? (1 = not at all, 5 = extremely). Bold text indicates a statistically significant difference. Median values are provided in parentheses. A five-point Likert scale (not at all = 1, slightly = 2, somewhat = 3, moderately = 4, extremely = 5) was used for each of these questions.

Survey Prompt	n	Initial Impression	Final Impression	*p*
Distance learning provides more motivation for acquisition of knowledge	38	1.5 ± 0.9 (1)	1.3 ± 0.6 (1)	0.103
There is no difference in the quality of knowledge acquired by distance learning and traditional lectures	38	2.0 ± 1.2 (2)	1.8 ± 1.1 (1.5)	0.250
Distance learning provides the possibility of independent evaluation	38	2.9 ± 1.4 (3)	3.1 ± 1.2 (3)	0.213
Distance learning provides independence of time and place for instruction	38	4.2 ± 0.9 (4)	4.2 ± 1.0 (4)	0.585
Distance learning requires possessing special skills to work on the computer	37	1.8 ± 0.9 (2)	1.6 ± 0.8 (1)	0.013
Face to face contact is necessary for acquiring and mastering the material	38	3.0 ± 1.3 (3)	3.1 ± 1.4 (3)	0.317
Distance learning provides faster and easier mastery of the material	37	1.8 ± 1.0 (1.5)	1.8 ± 1.2 (1)	1.0
Face to face contact is necessary for interactions with other students in the course	38	3.0 ± 1.3 (3)	3.1 ± 1.4 (3)	0.236

## Data Availability

The data presented in this study are contained within the article and supplemental materials.

## Supplementary Materials

The following are available online at https://www.mdpi.com/2306-7381/8/2/13/s1, Table S1: Outline of in-person lectures included in the 2019 face to face toxicology course. Materials covered in the final exams began with lecture 13, Table S2: Outline of topics included in the 2020 online toxicology course. Items identified with blue shading were considered supplemental materials. Materials covered on the Midterm and final exams began with Topics 4 and 49, respectively.
