# Supplementary material for: Effect of Delivery Format on Student Outcomes and Perceptions of a Veterinary Medicine Course: Synchronous Versus Asynchronous Learning
Source: Vet Sci. 2021 Jan 20;8(2):13. doi: 10.3390/vetsci8020013 (PMC7909406; doi:10.3390/vetsci8020013)
Supplement: Supplementary file 1 [file vetsci-08-00013-s001.pdf]

Effect of Delivery Format on Student Outcomes and Perceptions of a Veterinary Medicine Course: Synchronous versus Asynchronous Learning

Supplementary Data

Table S1. Outline of in-person lectures included in the 2019 synchronous toxicology course. Materials covered on the final exams began with lecture 13.

Lecture	Main topic
Lecture 1	Introduction to course; taking the toxicologic history
Lecture 2	Dose-response relationships; using toxicity data
Lecture 3	Diagnostic laboratory and introduction to treatment
Lecture 4	Management continued
Lecture 5	Management continued
Lecture 6	Neurotoxic poisonous plants
Lecture 7	Neurotoxic poisonous plants continued. Neurotoxic pesticides.
Lecture 8	Bromethalin and lead. Introduction to other metals.
Lecture 9	Lead poisoning. Intro to toxic plants that affect the gastrointestinal tract
Lecture 10	Plants that affect the gastrointestinal tract continued (e.g., pokeweed, castor bean). Iron
Lecture 11	Zinc and copper (copper affects multiple organ systems - renal and hepatic)
Lecture 12	Toxic foods (small animals)
Lecture 13	Feed related toxicants that affect livestock: urea/NPN and nitrates/nitrites
Lecture 14	Feed related toxicants that affect livestock: ionophores and salt
Lecture 15	Cardiac glycosides and start of cyanogenic plants
Lecture 16	Cyanogenic plants/cyanide continued. Start of toxic gases
Lecture 17	Toxic gases continued. Acute Bovine Pulmonary Edema and Emphysema
Lecture 18	Agents that target blood (e.g., anticoagulant rodenticides). Begin renal toxicants (pigweed).
Lecture 19	Renal toxicants continued (oak, lily, ethylene glycol)
Lecture 20	Renal toxicants continued (ethylene glycol continued, cholecalciferol).
Lecture 21	Teratogens. Start hepatotoxicants.
Lecture 22	Hepatotoxic agents: Death cap mushrooms, *Crotalaria spp*, aflatoxins
Lecture 23	Hepatotoxic agents continued: Cycads. Photosensitizers (primary vs. secondary). Venomous animals.
Lecture 24	Miscellaneous Top 10 Agents for Dogs and Cats: NSAIDs, acetaminophen, fipronil

Table S2. Outline of topics included in the 2020 asynchronous toxicology course. Items identified with blue shading were considered supplemental materials. Materials covered on the Midterm and final exams began with Topics 4 and 49, respectively.

Topic #	Title	Approximate Length (min:sec)
	**Introduction to the Course and Veterinary Toxicology**	
1	Greetings from Finland	7:15
2	Introduction to Course	6:50
3	Careers in Veterinary Toxicology	9:40
	**Unit I: Concepts in Veterinary Toxicology**	
4	Taking the Toxicological History	8:25
5	Dose Response Relationships	12:20
6	Sources of Toxicity Data	11:15
7	Factors that Influence Toxicity	8:15
8	Hazard versus Risk	5:10
	**Unit II: General Approaches to Diagnosis and Management of Toxicoses**	
9	Introduction to Veterinary Forensics (Toxicology)	11:20
10	The Toxicological Necropsy	4:30
11	The Toxicological Field Investigation	8:15
12A	How Confident Are You With Your Diagnosis? Part A. Causality	9:20
12B	How Confident Are You With Your Diagnosis? Part B. Application to Clinical Toxicology	8:10
12C	How Confident Are You With Your Diagnosis? Part C. Practice Examples	11:50
13	Management of Corrosive Exposures	14:20
14	Emetics	12:25
15	Gastric and Rumen Lavage	8:25
16	Adsorbents and Cathartics	12:35
17	Direct Removal of an Ingested Toxicant	3:25
18A	Direct Removal of an Ingested Toxicant. Part A. Ion Trapping	7:35
18B	Direct Removal of an Ingested Toxicant. Part B. Diuresis and Extracorporeal Methods	6:55
18C	Direct Removal of an Ingested Toxicant. Part C. Lipid Therapy	5:15
18D	Direct Removal of an Ingested Toxicant. Part D. Chelation	9:00
19	Antidotes	5:20
20	Dermal and Ocular Exposures	6:00
	**Unit III: Introduction to Organ Systems Toxicology**	
21	Veterinary Toxicology Textbooks	5:05
22	Mechanisms of Action	6:35
23	Dose-Dependent Transitions	6:45
24	Conditions of Plant Poisoning	13:05
25	Introduction to Organ System Toxicology	
25A	Introduction to Neurotoxicology	15:45
25B	Introduction to Renal Toxicology	14:45

25C	Introduction to Hepatic Toxicology	16:25
25D	Introduction to Inhalation Toxicology	20:05
25E	Introduction to Cardiovascular Toxicology	32:55
25F	Introduction to Reproductive Toxicology	20:05
25G	Introduction to Developmental Toxicology	16:15
25H	Introduction to Carcinogenicity	28:25
	**Unit IV: Neurotoxicants**	
26	Nicotine	15:05
27	Marijuana	13:50
28	Yellow Star Thistle	6:05
29	Fumonisins	9:05
30	Larkspur	9:45
31	Locoweed	17:55
32A	Cholinesterase Inhibitors	22:25
32B	Cholinesterase Inhibitors: Case Example	6:25
33A	Pyrethrin and Pyrethroid Insecticides	14:15
33B	Pyrethrin and Pyrethroid Insecticides: Case Example	17:55
34A	Bromethalin	11:50
34B	Bromethalin: Case Examples	10:55
35	Lead	25:25
	**Unit V: Gastrointestinal Tract (GIT) Toxicants**	
36	Overview of GIT Plants (Philodendron, Amaryllis)	12:30
37	Pokeweed	6:50
38	Nightshades	13:10
39	Castor Bean	10:00
40	Iron	19:30
41	Zinc	16:00
42	Copper	19:25
	**Unit VI: Toxic Foods (Small Animal)**	
43	Chocolate	12:35
44	Avocado	7:55
45	Xylitol	11:30
46	Grape	10:15
47	Onion	10:35
48	Miscellaneous Toxic Foods	15:25
	**Unit VII: Food Animal Toxicants**	
Topic #	Title	Approximate Length (min:sec)
49	Nitrate	17:55
50	Urea	13:05
51	Ionophores	19:30
52	Salt Poisoning/Water Deprivation	21:40
53	Arsenic	34:30
	**Unit VIII: Cardiovascular and Blood Toxicants**	
54	Cardiac Glycosides	24:35
55	Andromedotoxin	8:35
56	Japanese Yew	10:05
57	Cyanide	23:25

58A	Anticoagulant Rodenticides (Use Trends)	4:20
58B	Anticoagulants (Clinical Toxicology)	22:50
59	Red Maple	10:40
60	Hymenoptera	14:45
61A	North American Venomous Snakes: Coral Snakes	6:35
61B	North American Venomous Snakes: Pit Vipers	23:40
62	Bracken Fern	10:30
63A	Radiation	29:40
63B	Radon	7:50
	**Unit IX: Respiratory Toxicants**	
64	Air Pollution	20:00
65	Hydrogen Sulfide	20:50
66	Ammonia	9:50
67	Carbon Monoxide	14:30
68	Smoke Inhalation	15:15
69	Teflon	10:30
70	Acute Bovine Pulmonary Emphysema and Edema	15:30
71	Zinc Phosphide	15:20
	**Unit X: Renal Toxicants**	
72	Pigweed	9:35
73	Halogeton	8:25
74	Oak	7:15
75	Lilies	6:00
76	Ethylene Glycol	20:10
77	Cholecalciferol	15:10
78	NSAIDs	17:20
79	Blister Beetle	11:45
80	Ochratoxin A	12:00
	**Unit XI: Teratogens**	
81	Veratrum	10:10
82	Fescue	20:10
	**Unit XII: Hepatotoxic and Phototoxic Agents**	
83A	Mushrooms (Identification)	11:30
83B	Mushrooms (Amanita Mushrooms)	10:00
84	Pyrrolizidine Alkaloids	14:45
85	Aflatoxin	21:00
86	Acetaminophen	12:25
87	Cycads	10:20
88	Blue Green Algae	19:40
89	Photosensitization	16:35
	**Unit XIII: Miscellaneous Topics**	
90A	Small Animal Top Ten: Fertilizer	4:15
90B	Small Animal Top Ten: ADHD Stimulant Drugs	12:55
